# Episode- and Hospital-Level Modeling of Pan-Resistant Healthcare-Associated Infections (2020–2024) Using TabTransformer and Attention-Based LSTM Forecasting

**DOI:** 10.3390/diagnostics15172138

**Published:** 2025-08-25

**Authors:** Nicoleta Luchian, Camer Salim, Alina Plesea Condratovici, Constantin Marcu, Călin Gheorghe Buzea, Mădalina Nicoleta Matei, Ciprian Adrian Dinu, Mădălina Duceac (Covrig), Eva Maria Elkan, Dragoș Ioan Rusu, Lăcrămioara Ochiuz, Letiția Doina Duceac

**Affiliations:** 1 Doctoral School of Biomedical Sciences, Faculty of Medicine and Pharmacy, “Dunărea de Jos” University of Galați, 47 Domnească Street, 800008 Galați, Romania; nicoletaluchian13@yahoo.com (N.L.); madalinaduceac@yahoo.ro (M.D.); 2 Faculty of Medicine, Ovidius University of Constanta, 1 Universității Street, Campus-Corp A, etaj 1, 900470 Constanța, Romania; salimcamer@yahoo.com; 3 Faculty of Medicine and Pharmacy, “Dunărea de Jos” University of Galați, 47 Domnească Street, 800008 Galați, Romania; madalina.matei@ugal.ro (M.N.M.); cdinu@ugal.ro (C.A.D.); cojocarumariaeva@yahoo.com (E.M.E.); letimedr@yahoo.com (L.D.D.); 4 National Institute of Research and Development for Technical Physics, IFT Iași, 700050 Iași, Romania; calinb2003@yahoo.com; 5 Clinical Emergency Hospital “Prof. Dr. Nicolae Oblu” Iași, 2 Ateneului Street, 700309 Iași, Romania; 6 Department of Environmental Engineering, Mechanical Engineering and Agritourism, Faculty of Engineering, “Vasile Alecsandri” University of Bacău, 600115 Bacău, Romania; drusu@ub.ro; 7 Faculty of Medicine and Pharmacy, “Grigore T. Popa” University of Medicine and Pharmacy Iași, 700115 Iași, Romania; lacramioara.ochiuz@umfiasi.ro

**Keywords:** *Acinetobacter* spp., pan-drug resistance, healthcare-associated infections (HAIs), TabTransformer, LSTM forecasting, antimicrobial stewardship

## Abstract

**Background**: Pan-drug-resistant (PDR) Acinetobacterinfections are an escalating ICU threat, demanding both patient-level triage and facility-wide forecasting. **Objective**: The aim of this study was to build a dual-scale AI framework that (i) predicts PDR status at infection onset and (ii) forecasts hospital-level PDR burden through 2027. **Methods**: We retrospectively analyzed 270 *Acinetobacter* infection episodes (2020–2024) with 65 predictors spanning demographics, timelines, infection type, resistance-class flags, and a 25-drug antibiogram. TabTransformer and XGBoost were trained on 2020–2023 episodes (*n* = 210), evaluated by stratified 5-fold CV, and externally tested on 2024 episodes (*n* = 60). Metrics included AUROC, AUPRC, accuracy, and recall at 90% specificity; AUROC was optimism-corrected via 0.632 + bootstrap and DeLong-tested for drift. SHAP values quantified feature impact. Weekly PDR incidence was forecast with an attention–LSTM model retrained monthly (200 weekly origins, 4-week horizon) and benchmarked against seasonal-naïve, Prophet, and SARIMA models (MAPE and RMSE). Quarterly projections (TFT-lite) extended forecasts to 2027. **Results**: The CV AUROC was 0.924 (optimism-corrected 0.874); an ensemble of TabTransformer + XGBoost reached 0.958. The 2024 AUROC fell to 0.586 (*p* < 0.001), coinciding with a PDR prevalence drop (75→38%) and three covariates with PSIs > 1.0. Isotonic recalibration improved the Brier score from 0.326 to 0.207 and yielded a net benefit equivalent to 26 unnecessary isolation-days averted per 100 ICU admissions at a 0.20 threshold. SHAP highlighted Ampicillin/Sulbactam resistance, unknown acquisition mode, and device-related infection as dominant drivers. The attention–LSTM achieved a median weekly MAE of 0.10 (IQR: 0.028–0.985) vs. 1.00 for the seasonal-naïve rule, outperforming it on 48.5% of weeks and surpassing Prophet and SARIMA (MAPE = 6.2%, RMSE = 0.032). TFT-lite projected a ≥ 25% PDR tipping point in 2025 Q1 with a sustained rise in 2027. **Conclusions**: The proposed framework delivers explainable patient-level PDR risk scores and competitive 4-week and multi-year incidence forecasts despite temporal drift, supporting antimicrobial stewardship and ICU capacity planning. Shrinkage and bootstrap correction were applied to address the small sample size (EPV = 2.1), which poses an overfitting risk. Continuous recalibration and multi-center validation remain priorities.

## 1. Introduction

### 1.1. Antimicrobial Resistance in ICUs

Antimicrobial resistance (AMR) has emerged as a defining challenge in modern critical care, particularly in intensive care units (ICUs), where patients experience high exposure to invasive procedures, prolonged hospitalization, and frequent use of broad-spectrum antibiotics. Gram-negative organisms, such as *Acinetobacter baumannii* and *Klebsiella pneumoniae*, have shown alarming increases in carbapenem resistance (CR), multidrug resistance (MDR), and extended-spectrum β-lactamase (ESBL) production [1,2,3]. In some high-burden hospital settings, up to 80% of *Acinetobacter* isolates may be non-susceptible to carbapenems [4].

Pan-drug resistance (PDR), defined as non-susceptibility to all agents in all antimicrobial categories tested [5], represents the most extreme resistance phenotype. PDR infections in ICUs are associated with mortality rates exceeding 40–60%, particularly in cases of ventilator-associated pneumonia and bloodstream infections [6,7]. These cases also drive elevated costs and strain healthcare resources by requiring last-resort antibiotics such as colistin and cefiderocol, along with heightened infection-control measures [8].

### 1.2. The Need for Dual-Scale Modeling

Mitigating the impact of pan-resistance requires complementary decision-making tools at both the patient and institutional levels:

- Episode-Level Prediction: Clinicians must rapidly identify which infections are likely to be pan-resistant at the point of detection. Early alerts can support escalation to appropriate therapies before susceptibility results become available [9,10].
- Hospital-Level Forecasting: Infection control teams and administrators require forward-looking estimates of AMR trends to guide procurement, allocate ICU resources, and plan antimicrobial stewardship efforts [11].

Traditional antibiogram surveillance (e.g., EUCAST reports) is retrospective and descriptive. While valuable, these summaries do not offer predictive insights into the direction or timing of resistance surges. There is a pressing need for dynamic, predictive frameworks that integrate patient-level and population-level AMR data.

### 1.3. Existing Work and Remaining Gaps

Prior AMR modeling efforts have typically focused on a single scale. Episode-level models often rely on logistic regression or decision trees built from a limited set of clinical variables [12,13,14]. While interpretable, these methods may underperform when high-dimensional categorical features (e.g., 25-drug antibiograms) or missing data are present.

At the hospital level, forecasting studies have used ARIMA, SARIMA, and Prophet to model resistance trends over time [15,16]. However, these classical time-series approaches assume stationarity and fixed seasonality, limiting adaptability to dynamic hospital environments.

Recent studies (2022–2024) have begun exploring deep learning models for hospital-level infection forecasting, using LSTM or transformer encoders to account for non-linear temporal patterns [17,18]. Yet no prior study, to our knowledge, has combined transformer-based architectures for both

1. Patient-level PDR classification using embedded categorical inputs (e.g., species and antibiograms);
2. Multi-horizon hospital-level forecasting using attention-based temporal models.

### 1.4. Our Contributions

To address these gaps, we present a dual-scale modeling framework for pan-resistant *Acinetobacter* infections in a tertiary ICU (2020–2024). Our contributions are as follows:

1. Episode-Level Modeling: We used the TabTransformer architecture [19] to model structured EHR and LIS features, including demographics, ward location, infection type (IAAM), species flags, and 25-drug antibiograms. TabTransformer was selected for its ability to learn complex interactions among high-cardinality categorical variables. We compare its performance to XGBoost and interpret predictions using SHAP.
2. Hospital-Level Forecasting: We developed a Temporal Fusion Transformer-inspired model (TFT-lite) [20], implemented as an LSTM with an attention mechanism and seasonal encoding. This model was benchmarked against Prophet and SARIMA. The choice of LSTM-based attention models reflects their superior performance in capturing non-linear, non-stationary patterns in limited hospital time series.
3. Modeling-Ready Dataset: We cleaned and structured LIS export data into 270 microbiologically confirmed infection episodes, each with 65 features, including time-to-infection and resistance classifications.
4. Label Definition: PDR labels were assigned based on Magiorakos et al. (2012) [5], with “not tested” values imputed as susceptible to ensure consistent labeling from the 25-drug antibiogram.

**Figure 1:**
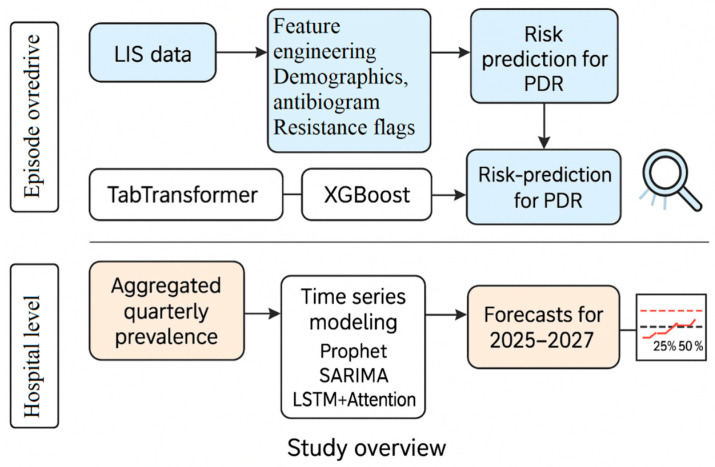
The study integrates episode-level and hospital-level modeling to predict and forecast pan-drug resistance (PDR). At the episode level, laboratory and clinical features are processed for PDR risk prediction using TabTransformer, XGBoost, and ensemble models. At the hospital level, quarterly PDR prevalence is aggregated and forecasted using time-series models (Prophet, SARIMA, and LSTM with attention). Results inform both patient-specific risk and institutional planning across 2025–2027.

To our knowledge, this is the first study to implement dual transformer-based models for both real-time pan-resistance prediction and forward-looking prevalence forecasting. Figure 1 summarizes the study design.

The remainder of this paper is organized as follows: Section 2 describes the dataset, preprocessing pipeline, and modeling methods used for both the episode-level classification and hospital-level forecasting. Section 3 presents the experimental results, including classifier performance, SHAP-based interpretability, and forecasting accuracy across models. Section 4 discusses clinical and operational implications, strengths, and limitations of the dual-scale framework. Finally, Section 5 concludes with future directions for external validation, genomic integration, and deployment of predictive dashboards in real-time hospital workflows.

## 2. Materials and Methods

### 2.1. Dataset and Feature Engineering

**Figure 2:**
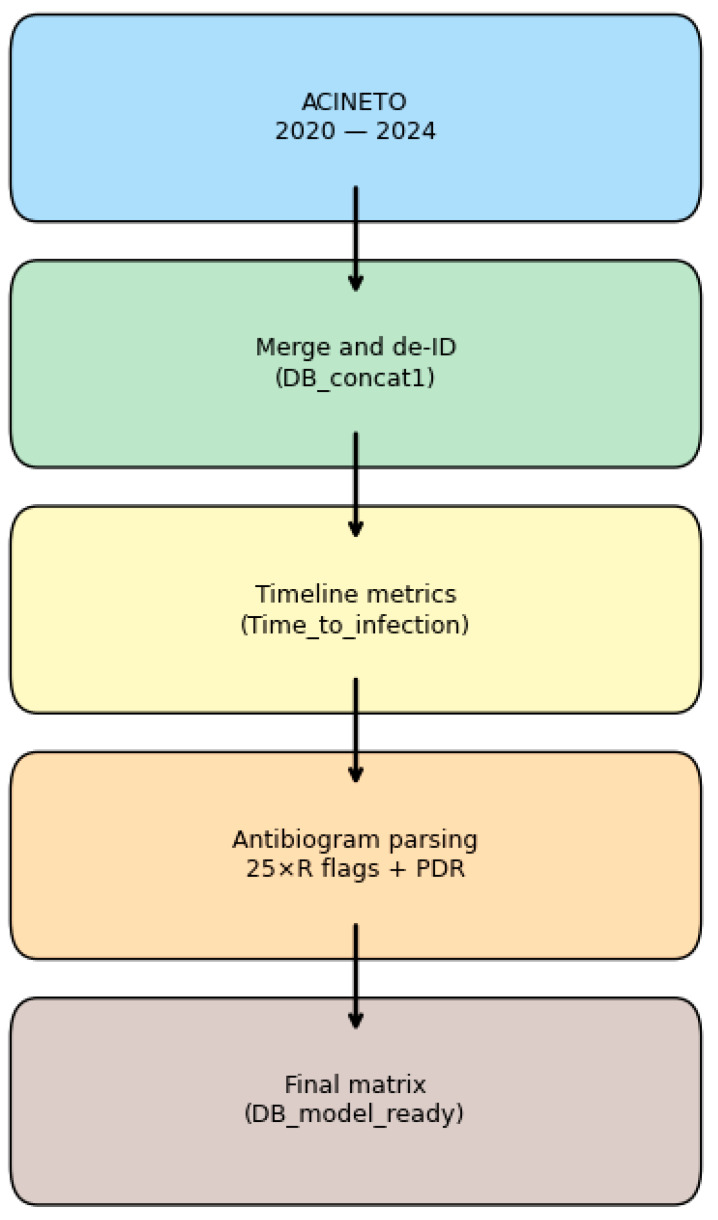
Data-preparation workflow from raw LIS exports to modeling matrix.

This section outlines the data pipeline from raw hospital laboratory exports to a finalized, machine learning-ready dataset. It details the source, cohort, preprocessing, feature engineering, and matrix construction. Figure 2 presents a schematic workflow, and Table 1 and Table 2 summarize dataset properties.

#### 2.1.1. Data Source and Cohort Description

We used data from hospital microbiology and clinical records spanning 1 January 2020 to 31 December 2024. Each record represents a microbiologically confirmed *Acinetobacter* spp. infection episode from an ICU or non-ICU ward.

- Inclusion Criteria: adult patients with confirmed *Acinetobacter* isolate and a complete antibiogram.
- Total Episodes: 270, distributed as follows: 2020: 32 episodes; 2021: 54 episodes; 2022: 39 episodes; 2023: 52 episodes; 2024: 93 episodes.

**Figure 3:**
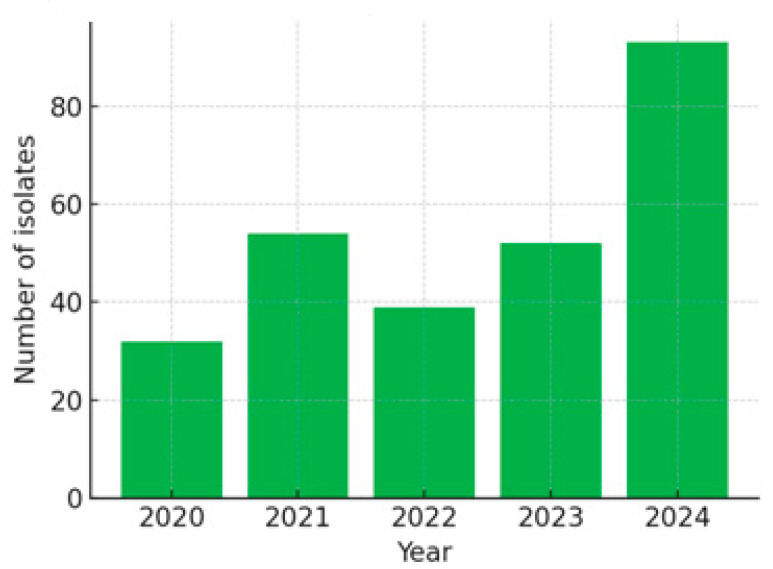
Annual distribution of infection episodes (bar chart, 2020–2024).

*Acinetobacter* spp. were selected due to their known high rates of resistance and impact in ICU infections. Annual LIS exports containing ~20 variables per episode were concatenated (DB_concat1, 270 rows × 13 columns), with direct identifiers and free-text diagnosis fields removed (see Table 1). Figure 3 illustrates the yearly distribution.

#### 2.1.2. Timeline Features

Admission Date and Infection Detection Date were formatted using a consistent date structure in the form of year–month–day (YYYY-MM-DD) to ensure uniformity in temporal data representation. Time to Infection (in days) was derived from their difference. Two records (0.74%) had missing values, which were imputed using the cohort median (7 days). Timestamp consistency across years was validated to ensure correct interval calculations.

#### 2.1.3. Antibiogram Parsing and Resistance Labels

Each record contained three semicolon-separated susceptibility lists (S/I/R) for 25 antibiotics. After parsing:

- Each drug was encoded as: 1 = Susceptible, 0.5 = Intermediate, 0 = Resistant.
- Non-tested values (e.g., NT or −1) were conservatively imputed as 1 (Susceptible) to avoid false inflation of resistance estimates. This assumption prioritizes specificity but may under-represent true resistance in some cases.
- Pan-drug resistance (PDR) was defined as per Magiorakos et al. (2012) [5] as resistance to all tested agents. Using this definition, 169 of 270 episodes (62.6%) were labeled as PDR.

#### 2.1.4. Feature Engineering

Romanian column headers were translated and organized into a structured schema. The final matrix included 65 engineered features:

Demographics and Ward Assignment

- Sex (binary), Age (years), Ward (ICU/non-ICU), and Ward Dummies.

Infection Type and Pathogen

- Infection_Type, Identified_Pathogen, is_A_calcoaceticus, and is_A_lwoffi.

Resistance Class Flags

- Is_RC, is_MDR, and is_ESBL (binary).

Timeline Features

- Admission_Date, Infection_Detection_Date, Length_of_Stay, and Time_to_Infection.

Antibiotic Susceptibility

- Twenty-five antibiotic columns (AB_1 to AB_25), each encoded as follows: 1 = Susceptible, 0.5 = Intermediate, 0 = Resistant; AB_1—AMIKACIN through AB_25—NITROFURANTOIN.

Outcome

- Pan_resistant: binary outcome (1 = PDR).

Temporal Metadata

- Year: integer (2020–2024).

Categorical fields were one-hot-encoded; numerical fields were imputed using the cohort median where missing. The final modeling matrix (DB_model_ready.csv) included 270 rows × 65 columns, with no missing values. The PDR label was reproducible directly from the antibiogram flags.

### 2.2. Exploratory Data Analysis

#### 2.2.1. Class Distribution and Handling of Imbalance

Of the 270 total *Acinetobacter* infection episodes included in the cohort, 169 (62.6%) were labeled pan-resistant (PDR = 1), while 101 (37.4%) were non-pan-resistant (PDR = 0). This resulted in a moderately imbalanced dataset, in which the positive class (PDR) was unexpectedly the majority, contrary to typical medical classification problems.

This unusual class distribution has two important methodological implications:

1. Evaluation Metrics: Because overall accuracy can be misleading in imbalanced datasets, we emphasize threshold-independent metrics such as AUROC and AUPRC, along with recall and F1-score, to fairly evaluate model performance.
2. Class Handling in Training: All cross-validation folds are stratified on the pan_resistant label to preserve class proportions. Where algorithmically supported, inverse-frequency class weights are applied to penalize misclassification of the minority class. In sensitivity analyses, we also evaluate the impact of SMOTE (Synthetic Minority Over-Sampling Technique) to address class imbalance.

**Figure 4:**
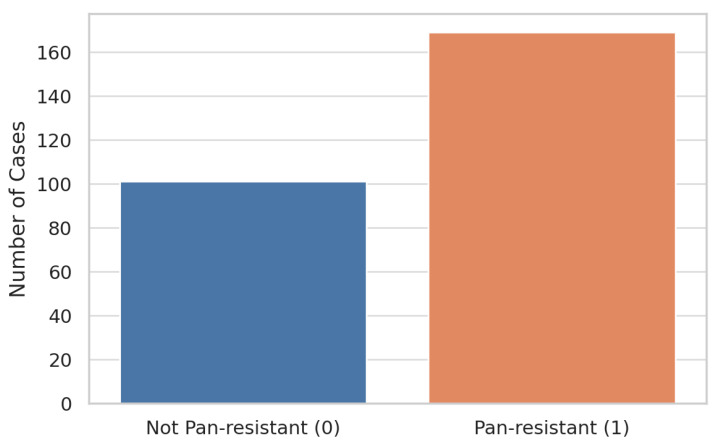
Bar plot of pan-resistant (PDR = 1) versus non-pan-resistant (PDR = 0) episodes in the study cohort.

Figure 4 displays a bar plot summarizing the class distribution.

#### 2.2.2. Numeric Feature Distributions

We examined three continuous variables—Age, Time to Infection, and Length of Stay (LOS)—to characterize their distributions and evaluate their potential predictive contribution.

- All three variables exhibit positive skewness (right-tailed), a typical pattern in clinical datasets involving hospitalization duration.
- Age spans from 2 to 93 years (mean: 55.4, median: 57.5), with a concentration in the 55–65-year range, consistent with the age distribution of ICU patients.
- Time to Infection (days from admission to positive culture) and LOS show similar distributions, both peaking at 10–15 days and tapering into long tails extending beyond 90 days.
- Notably, 8 episodes (2.9%) had a Time to Infection = 0 days, potentially representing community-acquired infections or data entry errors. These were retained as their impact on distribution and modeling was minimal.
- A strong positive correlation was observed between LOS and Time to Infection (Spearman’s ρ = 0.73, *p* < 0.001), suggesting that they largely track the same clinical timeline. This correlation raises considerations around multicollinearity in regression models.

All three variables are integer-valued, contain no missing values, and will be z-standardized before being included in predictive models.

**Figure 5:**
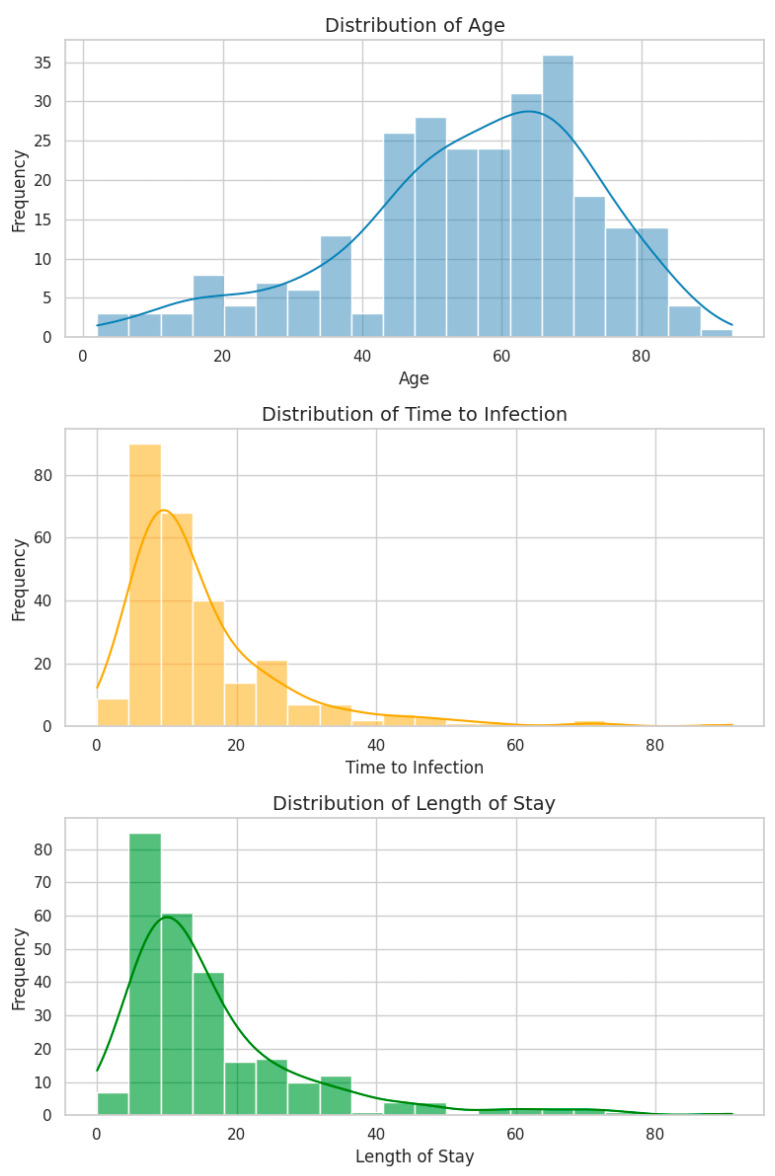
Histograms with KDE curves for Age, Time to Infection, and Length of Stay. Each distribution is right-skewed; most infections occurred within the first 20 days of hospitalization.

Figure 5 displays histograms with kernel density overlays for each variable, while Table 3 provides descriptive statistics, including means, standard deviations, quartiles, and ranges.

#### 2.2.3. Resistance Flags and Antibiotic Profiles

To explore relationships between the outcome variable and the 25 individual antibiotic features, we computed a Pearson correlation matrix including the outcome (pan_resistant), resistance-class flags (is_RC, is_ESBL, and is_MDR), and all drug-specific resistance indicators.

**Figure 6:**
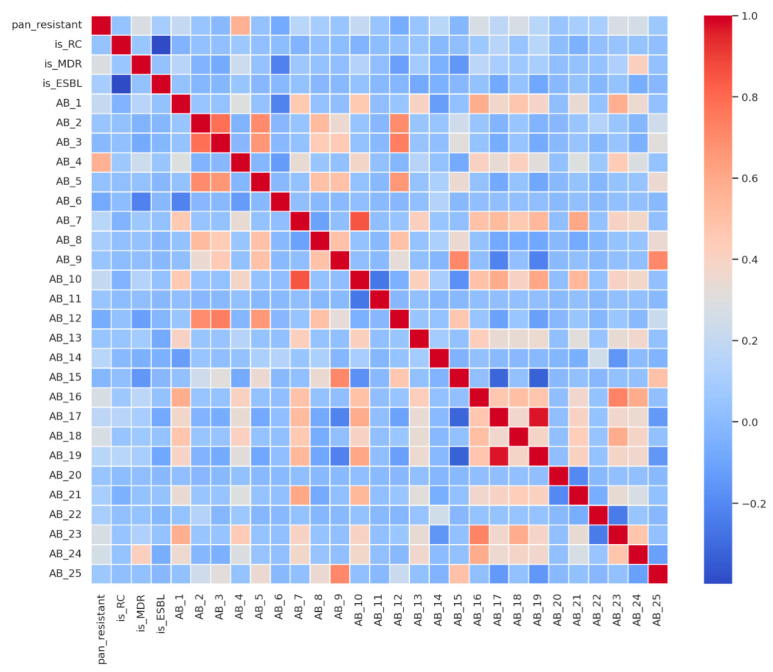
Correlation heatmap of outcome and resistance features.

Key findings from Figure 6 (heatmap) include the following:

- Nearly all antibiotics show negative correlations with PDR, indicating that retained susceptibility (value = 1) is inversely related to pan-resistance. This is biologically expected and statistically reassuring.
- AB_4 stands out with a positive correlation with the pan_resistant label, raising concern about either mislabeling or a biologically atypical pattern. This feature will be specifically audited before modeling.
- Summary flags is_RC (resistance class) and is_ESBL show near-zero correlation with the outcome due to their low variance in the dataset—RC is nearly universal (~98%), and ESBL is rare (~1.5%).
- Strong intra-class correlations are observed among antibiotics from the same pharmacologic groups (e.g., carbapenems and aminoglycosides), reflecting known patterns of cross-resistance in *Acinetobacter* spp.

Implications for modeling include the following:

1. Individual antibiotic resistance flags appear more predictive of pan-resistance than high-level summary indicators like is_MDR or is_RC, which show low variability and low signal-to-noise ratios.
2. Multicollinearity among antibiotics (particularly within classes) necessitates control through Elastic-net regularization for linear models and the use of tree-based methods (e.g., gradient boosting), which are more robust to correlated features.

#### 2.2.4. Univariate Associations with Pan-Resistance

We assessed the individual association of each predictor with the binary outcome variable (pan_resistant = 1 vs. 0). Continuous variables (Age, Time to Infection, and Length of Stay) were compared using the two-sided Mann–Whitney U test. Categorical predictors and binary antibiotic resistance flags were analyzed using either Fisher’s exact test or χ^2^ tests, as appropriate.

To correct for multiple hypothesis testing across many features, we applied the Benjamini–Hochberg false discovery rate (FDR) adjustment. Results are summarized in Table 4 (numeric) and Table 5 (categorical + antibiotic flags).

- None of the numeric variables demonstrated a statistically significant difference between the PDR and non-PDR groups after adjustment (q > 0.05).
- Among the categorical and antibiotic features, nine antibiotics showed statistically significant associations with PDR (q < 0.05), with the strongest signals arising from carbapenems (e.g., AB_16 and AB_18), fluoroquinolones (e.g., AB_4), and aminoglycosides (e.g., AB_23).
- One infection-type category (IAAM_UNKNOWN) also retained significance and will be included in downstream multivariate analysis.

These features form the basis of the initial candidate set for model input, pending checks for multicollinearity.

#### 2.2.5. Multicollinearity in the Antibiotic Block

Given the dense antibiotic feature set, we assessed multicollinearity to identify redundant predictors and inform regularization strategies:

- Low-variance filtering removed four binary variables with near-zero variance: is_A_calcoaceticus, is_RC, is_ESBL, and is_A_lwoffi. These features contribute minimal signals and can inflate model variance.
- We computed Variance Inflation Factors (VIFs) for the 21 remaining antibiotic resistance features (coded as 1 = resistant, 0 = susceptible). Twelve features exceeded the conventional VIF > 10 threshold, with AB_9 showing extreme multicollinearity (VIF ≈ 1300) (see Table 6).
- The highest VIFs were concentrated among carbapenems and β-lactams, confirming known pharmacologic cross-resistance and redundancy.

Modeling approach: We retained these clinically meaningful variables while mitigating instability through the following:

1. Elastic-net regularization in linear models (e.g., logistic regression);
2. Tree-based ensemble methods (e.g., XGBoost), which are robust to multicollinearity;
3. Sensitivity analysis using grouped drug-class features to assess robustness.

#### 2.2.6. Latent Structure of Resistance

We applied Principal Component Analysis (PCA) to the 21 binary antibiotic resistance features (1 = resistant, 0 = susceptible) to explore latent structure and dimensionality.

- The first principal component (PC1) accounted for 35.3% of total variance and strongly captured a global resistance gradient, effectively separating PDR from non-PDR cases.
- PC2 explained 11.9% of variance and reflected secondary co-resistance patterns but contributed limited additional discrimination.

**Figure 7:**
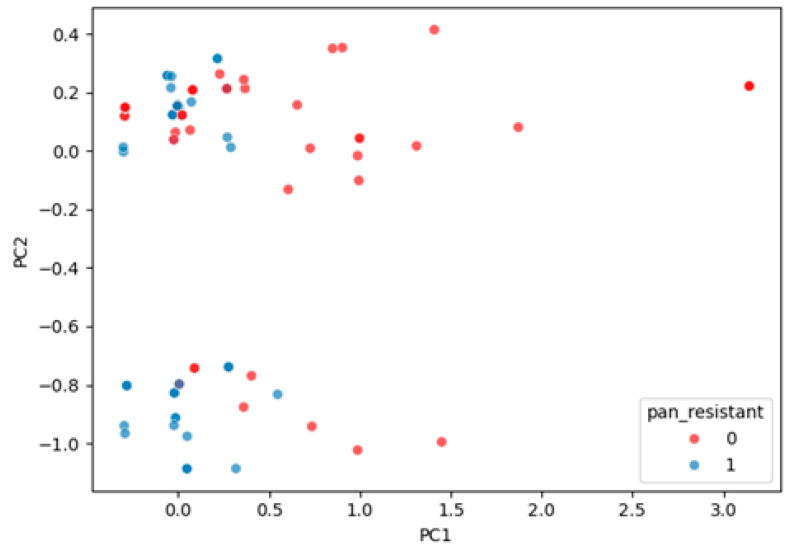
PCA scatter plot of the 21 antibiotic resistance flags. Points are colored according to pan-resistance (blue = PDR, red = non-PDR). PC1 (35% of variance) reflects an overall resistance gradient; PC2 (12% of variance) adds a weaker secondary pattern.

The visualization in Figure 7 shows clear separation of PDR vs. non-PDR groups along PC1, validating its relevance for classification.

#### 2.2.7. Temporal Drift

**Figure 8:**
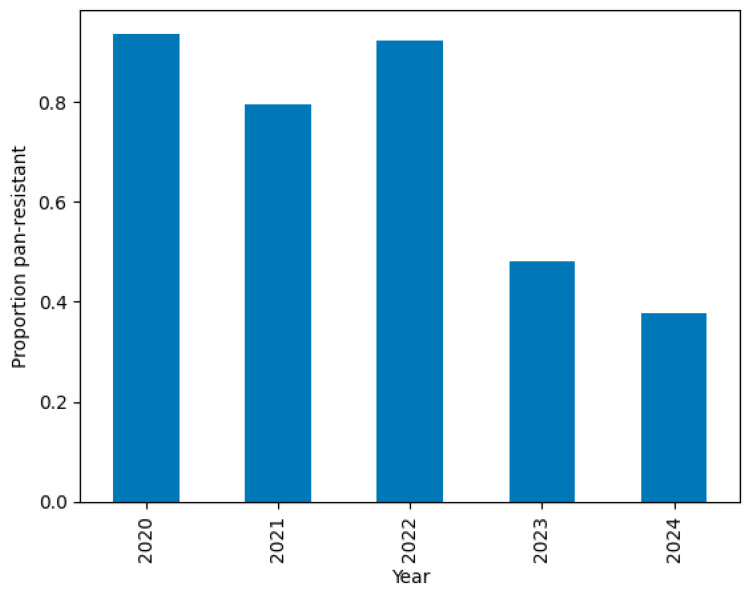
Yearly prevalence of PDR with 95% confidence intervals.

To evaluate potential shifts in resistance prevalence over time, we examined annual trends in the proportion of PDR cases (Figure 8). A Cochran–Armitage trend test indicated a significant downward trend in PDR incidence across the five-year period (χ^2^ = 9.6, *p* = 0.002).

- PDR prevalence was >80% from 2020 to 2022 but dropped to 48% in 2023 and 38% in 2024.
- This temporal drift motivates the segregation of 2024 as a temporal hold-out set, used exclusively for external validation.

#### 2.2.8. Baseline Discriminative Performance

As a benchmark for more complex models, we trained a logistic regression classifier on the development set (2020–2023).

- Continuous features were z-standardized, and binary features were retained as-is.
- L2 regularization was applied using the saga optimizer, and class imbalance was addressed with inverse class weights.
- Five-fold stratified cross-validation yielded a mean AUROC ≈ 0.75 ± 0.11, establishing a baseline performance threshold.

This result reflects a strong starting point given that no feature selection or hyperparameter tuning was applied. Future models must exceed this threshold to justify additional complexity.

#### 2.2.9. Antibiogram Patterns by Species, Ward, and Year

To complement statistical analyses, we visualized patterns in resistance by stratifying the antibiogram across temporal and clinical subgroups.

**Figure 9:**
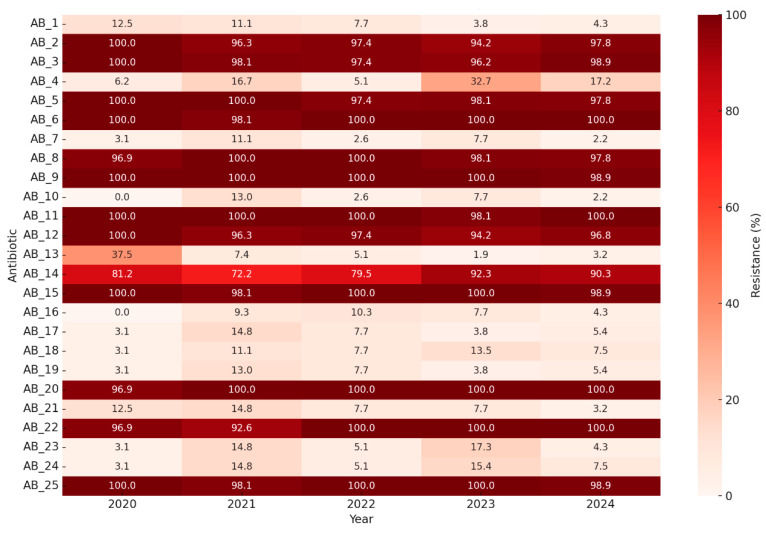
Year-by-year heatmap of antibiotic resistance percentages.

**Figure 10:**
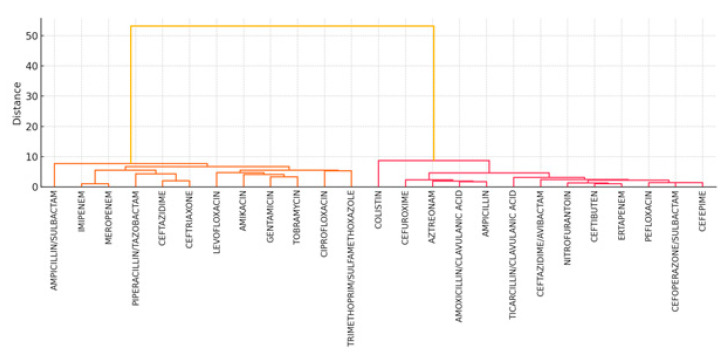
Ward’s linkage clustering dendrogram of 25 antibiotics.

- Figure 9 presents a heatmap of antibiotic resistance rates across calendar years. Resistance levels remain consistently high, particularly among carbapenems, but some fluctuations in specific antibiotics (e.g., meropenem and tobramycin) may reflect empirical prescribing changes or strain replacement.
- Figure 10 shows unsupervised hierarchical clustering of the 25 antibiotics. Resulting dendrograms support known pharmacologic groupings (e.g., aminoglycosides and fluoroquinolones) and reinforce the earlier findings of intra-class correlation and redundancy.

These visual insights support the rationale for preserving full antibiotic profiles with appropriate regularization.

### 2.3. Episode-Level Prediction Models

This section outlines the machine learning approaches used to predict pan-drug resistance (PDR) at the time of infection episode detection. The objective is to benchmark multiple modeling paradigms that balance predictive performance with interpretability.

Three complementary classifiers were selected:

- TabTransformer (transformer-based deep learning),
- XGBoost (gradient-boosted decision trees), and
- Soft Voting Ensemble (probabilistic averaging of TabTransformer and XGBoost).

These methods were chosen to represent diverse modeling families—deep neural networks, decision tree ensembles, and hybrid approaches—providing a robust basis for comparison.

#### 2.3.1. Modeling Strategies and Technical Details

We benchmarked three supervised classification approaches to assess the feasibility of early PDR prediction from structured electronic health record (EHR) features. These methods reflect three distinct modeling paradigms—deep learning, decision tree ensembles, and model averaging—selected to balance expressiveness, interpretability, and sample efficiency (see Shateri et al. [21]):

1. TabTransformer is a transformer-based neural architecture tailored for tabular data, particularly effective for high-cardinality categorical variables. It embeds each categorical column into a dense vector and passes the embeddings through self-attention blocks [19]. The key operation, scaled dot-product attention, is defined as follows:

(1) $$Attention\left(Q,K,V\right)=softmax\left(\frac{QK^{T}}{\sqrt{d_{k}}}\right)V$$

where *Q*, *K*, and *V* are learned query, key, and value matrices and *d_k_* is the embedding dimension. This architecture learns complex, context-aware interactions across input features.

2. XGBoost is a gradient-boosting algorithm that builds an ensemble of decision trees by sequentially optimizing a regularized objective. For a given instance, iii, the predicted probability is:

(2) $${\hat{y}}_{i}=\sigma \left(\sum_{k=1}^{K}f_{k}\left(x_{i}\right)\right)$$

where *f_k_* represents each regression tree and *σ*(⋅) is the sigmoid activation function. XGBoost is known for its robustness to missing values and strong performance on tabular datasets.

3. The soft voting ensemble combines the probabilistic outputs of TabTransformer and XGBoost through unweighted averaging:

(3) $${\hat{y}}_{ensemble}=\frac{1}{2}\left({\hat{y}}_{TabTransformer}+{\hat{y}}_{XGBoost}\right)$$

This ensemble approach aims to capture complementary strengths and reduce variance.

To improve interpretability, we used SHAP (SHapley Additive exPlanations) to decompose predictions into feature-level contributions:

(4) $$f\left(x\right)=\phi_{0}+\sum_{j=1}^{M}\phi_{j}$$

where *ϕ_j_* quantifies the contribution of feature *j* to the model’s output *f*(*x*) and *ϕ*_0_ is the expected value over the training data. SHAP enables both local (case-level) and global (population-level) insights.

**Figure 11:**
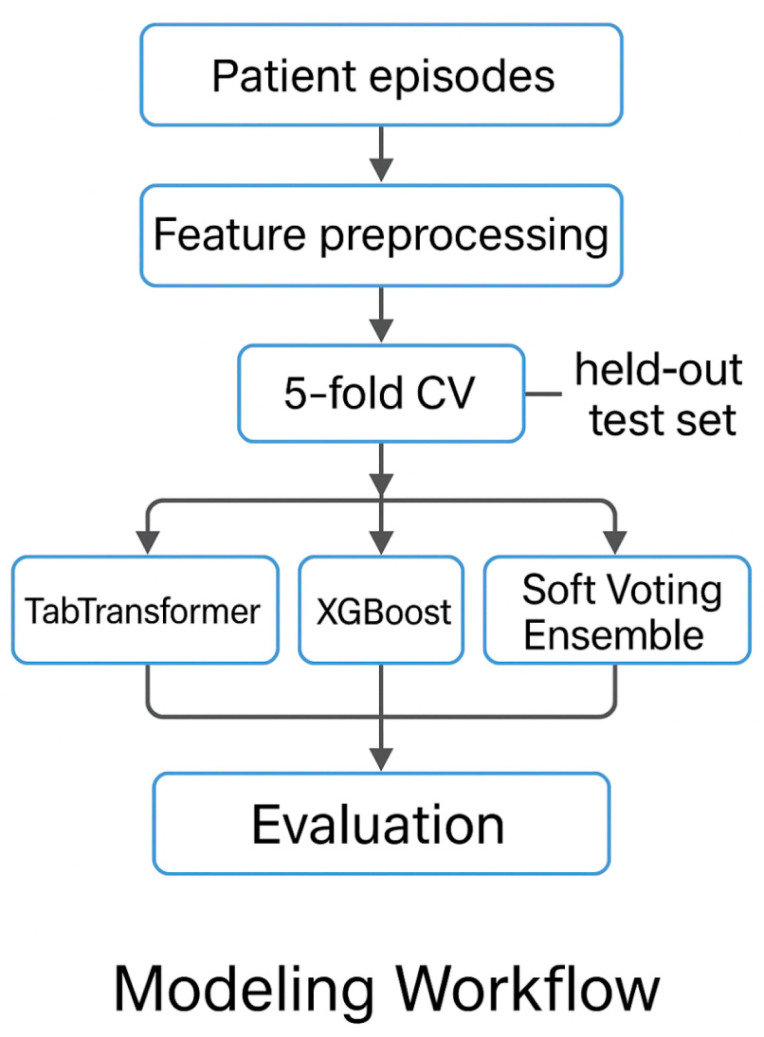
Overview of the episode-level modeling pipeline, including preprocessing, model training (2020–2023), and held-out evaluation on the 2024 cohort.

A schematic of the overall modeling pipeline is shown in Figure 11.

#### 2.3.2. Dataset Partitioning and Cross-Validation

To simulate prospective deployment and account for temporal distribution drift, we adopted a chronological split strategy:

- Training set (2020–2023): *n* = 210 episodes;
- Hold-out test set (2024): *n* = 60 episodes.

The training set was used for model development, hyperparameter tuning, and 5-fold stratified cross-validation. The 2024 cohort was reserved for final, out-of-sample evaluation.

#### 2.3.3. Feature Engineering and Preprocessing

The input feature matrix includes 65 engineered variables grouped into the following:

Categorical features:

- ICU ward assignment;
- Infection type (e.g., ITU, PAI, IIC, sepsis, or IRI);
- Species flags (e.g., *A. calcoaceticus* or *A. lwoffi*);
- Resistance class flags (is_MDR, is_ESBL, or is_RC);
- Antibiotic susceptibility indicators (25 binary features encoded as {0 = R, 0.5 = I, 1 = S}).

These were

- Embedded as tokens in TabTransformer;
- One-hot-encoded in XGBoost.

Continuous features: Age, Time to Infection, and Length of Stay.

These variables were z-score-standardized prior to model input. No missing values remained after preprocessing.

#### 2.3.4. TabTransformer Design

The TabTransformer architecture was implemented following Huang et al. (2020) [19] and adapted for binary classification:

- Embedding layer: 16-dimensional embeddings for each categorical feature;
- Transformer encoder: 2 layers and 8 attention heads;
- Feedforward head: 2 dense layers (ReLU activations), dropout = 0.3;
- Output layer: sigmoid activation for binary probability output.

The model was trained using:

- Optimizer: AdamW;
- Loss function: Binary Cross-Entropy;
- Regularization: Early stopping on validation AUROC.

The batch size and learning rate were tuned via grid search.

#### 2.3.5. XGBoost Configuration

The XGBoost baseline model was trained on the same training folds with numeric input:

- Categorical variables were fully one-hot-encoded;
- Antibiotic features were used as-is (values in {0, 0.5, 1});
- Continuous variables were standardized.

Key hyperparameters (max depth, learning rate, subsample ratio, and number of estimators) were optimized using a 5-fold grid search on training folds.

#### 2.3.6. Evaluation Protocol

Each model was evaluated using two-tier validation:

1. Internal validation via stratified 5-fold cross-validation on the 2020–2023 training set;
2. External validation on the 2024 hold-out test set (unseen during training).

Metrics used for model comparison included:

- Area under the receiver operating characteristic curve (AUROC);
- Area under the precision–recall curve (AUPRC);
- F1-score and accuracy;
- Recall at fixed specificity (90%), reflecting a clinically relevant alert threshold.

**Figure 12:**
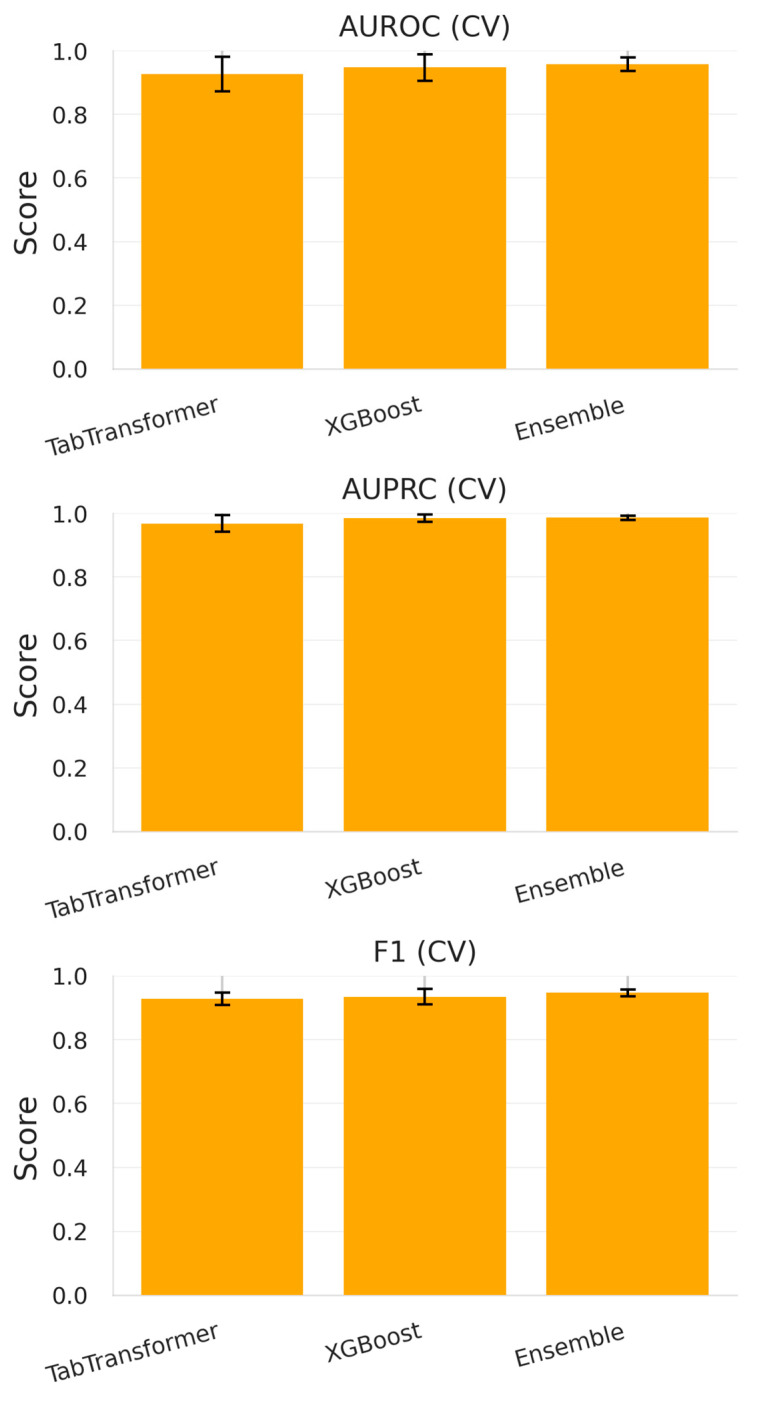
Bar plots of cross-validated metrics (AUROC, AUPRC, and F1-score) for each model (TabTransformer, XGBoost, and Ensemble) across the 2020–2023 training set.

**Figure 13:**
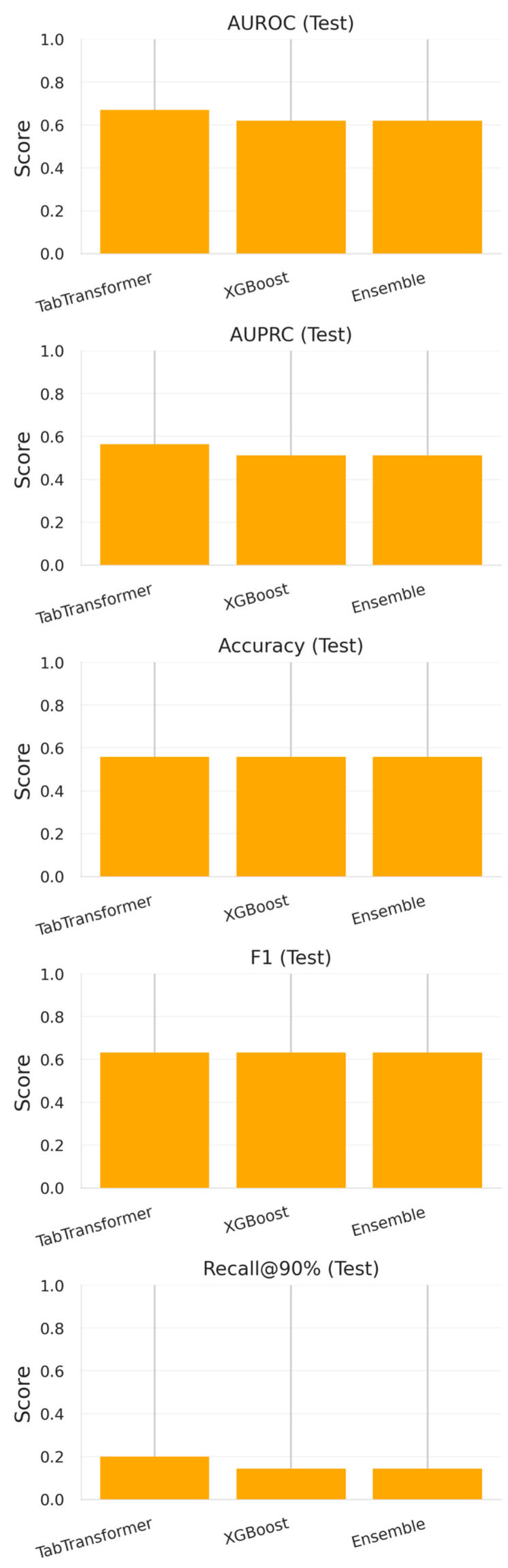
Bar plots of 2024 test set performance metrics for each model. Despite superior CV metrics, test performance was notably lower across models—suggesting temporal distribution shift.

Results are presented in Section 3.1 and Figure 12 and Figure 13.

#### 2.3.7. Implementation Details

All modeling and analysis were performed using Python 3.11 on Google Colab Pro+, a cloud-based platform provided by Google LLC, headquartered in Mountain View, California, United States. The runtime utilized an NVIDIA Tesla T4 GPU (16 GB VRAM) and 25 GB system RAM, provisioned dynamically via Google’s infrastructure. Details of the software stack are summarized in Table 7.

Model-training hyperparameters were tuned with five-fold stratified cross-validation on the 2020–2023 development set. Each experiment was run with a fixed random seed (seed = 42) to ensure exact reproducibility across reruns. Full source codes, notebooks, and a Dockerfile that recreates the above environment are available on request.

#### 2.3.8. Feature Attribution

To enable model interpretability and trust in clinical applications, we applied SHAP (SHapley Additive Explanations) to the trained TabTransformer. A permutation-based explainer was used to

- Quantify the marginal contribution of each feature to the predicted probability;
- Identify key clinical and laboratory drivers of PDR classification.

Full SHAP summary plots and feature rankings are provided in Section 3.3.

#### 2.3.9. Temporal Validation and Drift Analysis

Dataset split. All episodes from 2020 to 2023 (*n* = 177) comprised the development set; 2024 data (*n* = 93) served as an unseen temporal test cohort.

Internal performance. Discrimination during development was estimated with 5-fold stratified cross-validation; 95% confidence intervals (CIs) were computed by 2000-sample bootstrap resampling of out-of-fold predictions.

External performance. The final model, trained on the entire development set, was evaluated once on the 2024 cohort. The significance of the AUROC change was assessed with a 5000-label permutation test (null: no AUROC difference).

Covariate shift. Drift was quantified per feature using the Population Stability Index (PSI), flagging PSI > 0.20 as substantial, and multivariately with the Jensen–Shannon (JS) distance on the 25-dimensional antibiotic resistance vector.

Label shift. Changes in pan-drug-resistant (PDR) prevalence between periods were tabulated.

Mitigation experiment. To simulate quarterly updates, the first 20% of the 2024 records was appended to the training data, the model was retrained, and performance re-evaluated on the remaining 80%.

Predicted probabilities for 2024 were recalibrated with isotonic regression, and clinical usefulness was assessed by decision-curve analysis (thresholds: 0.05–0.50).

Sample-size and optimism correction. The development set contained 134 PDR events across 65 candidate predictors (events per variable = 2.1). Using the Riley et al. (2019) formula for binary prediction models, at least ~200 events would be required to limit overall shrinkage to ≤10%; our smaller event count therefore necessitated explicit optimism correction [22].

Apparent discrimination was adjusted via a 0.632+ bootstrap (200 resamples, balanced logistic regression, max_iter = 5000), yielding an optimism-corrected AUROC of 0.874.

To mitigate coefficient inflation we computed the global shrinkage factor (χ^2^ method, near-zero L2 penalty C = 10^6^), obtaining 0.696; predicted log-odds were therefore multiplied by 0.696 before probability calibration.

### 2.4. Hospital-Level Forecasting

This section describes the modeling strategy used to forecast quarterly prevalence of pan-drug-resistant (PDR) *Acinetobacter* infections at the hospital level. The forecasting objective is to anticipate future trends (2025–2026) using time-series models trained on quarterly data from 2020 to 2024. We evaluated both classical and deep learning-based approaches.

#### 2.4.1. Quarterly Aggregation and Feature Construction

To capture temporal trends in resistance, all infection episodes were aggregated by calendar quarter, yielding 20 time points (2020 Q1 to 2024 Q4). For each quarter, we computed the following:

- Resistant_count: the number of PDR-classified episodes;
- Total_count: the total number of episodes recorded;
- Prevalence: the proportion of pan-resistant infections, computed as resistant_count/total_count.

To enrich the time series with a seasonal and autoregressive structure, we engineered the following covariates:

- Seasonal Encoding: Applied sine and cosine transforms to the quarter index (cyclical encoding) to model periodic fluctuations.
- Lag Feature: Introduced lagged_prevalence [t − 1] to capture short-term autocorrelation in resistance rates.

These derived features were used consistently across all forecasting models.

#### 2.4.2. Prophet Forecasting Model (Baseline)

We implemented the Facebook Prophet model as a baseline classical forecaster. Prophet is a modular additive model that decomposes time series into trend, seasonal, and holiday components.

- Training window: 2020 Q1 to 2024 Q4 (20 time points);
- Forecast horizon: 8 quarters (2025 Q1 to 2026 Q4);
- Seasonality: configured to quarterly frequency using Fourier terms (order = 3);
- Trend modeling: automatic changepoint detection with default priors.

Prophet was selected for its interpretability and wide usage in epidemiological time-series tasks.

#### 2.4.3. SARIMA Forecasting

As a second baseline, we fitted a Seasonal AutoRegressive Integrated Moving Average (SARIMA) model:

- Model specification: SARIMA (1,0,0)(1,0,0) [4], incorporating annual seasonality (4 quarters/year).
- Transformation: Applied square-root transformation to the prevalence series to stabilize variance and satisfy normality assumptions.
- Model selection: Parameter tuning was guided by Akaike Information Criterion (AIC) minimization.
- Residual diagnostics: Autocorrelation and partial autocorrelation plots were inspected to confirm white noise residuals.

This classical time-series model provides a statistical reference point for evaluating more flexible neural approaches.

#### 2.4.4. LSTM with Attention (TFT-Lite) and Noise Injection

Given the short sequence length and limited sample size, we implemented a lightweight variant of the Temporal Fusion Transformer (TFT) [20], referred to as TFT-lite. This deep learning model incorporates both long short-term memory (LSTM) units and attention mechanisms for multi-horizon forecasting.

Model design:

- Input features: quarter index; seasonal encodings (sine/cosine of time index); lagged prevalence (t − 1);
- Sequence length: 11 past quarters used as input context;
- Target window: prevalence values forecasted for the next 8 quarters.

Architecture components:

- One-layer LSTM for temporal encoding;
- Multi-head attention layer for context weighting;
- Dense projection layer for output mapping;
- Sigmoid activation to constrain outputs between 0 and 1.

Training setup:

- Loss function: mean square error (MSE);
- Optimizer: Adam;
- Epochs: 200;
- Regularization: Gaussian label noise ε ∼ 𝒩(0, 0.05^2^) added to targets during training to mitigate overfitting.

This model was implemented in PyTorch and trained with early stopping based on validation loss.

#### 2.4.5. Rolling-Origin Weekly Evaluation

To assess short-term forecasting skill under realistic deployment constraints, we aggregated PDR episodes into Monday-anchored weekly counts (6 January 2020–29 December 2024; 256 weeks).

Forecasts were evaluated with an expanding-window walk-forward split: at each origin the model predicted the incidence four weeks ahead and was then advanced one calendar week.

A lightweight LSTM (8 hidden units, 5 epochs) was retrained at the start of each calendar month; the comparator was a seasonal-naïve rule (mean of the same weekday across the four preceding weeks).

Performance was summarized by mean absolute error (MAE) and root mean square error (RMSE); superiority was defined as a lower MAE at a given origin.

### 2.5. Ethics Statement

This study was approved by the Institutional Ethics Committee of “Prof. Dr. Nicolae Oblu” Clinical Emergency Hospital, Iași, Romania (Approval No. 2/23.02.2023; date of approval: 23 February 2023) with a waiver of informed consent because all data were retrospective and de-identified prior to analysis.

### 2.6. Code Availability

All anonymized scripts, including the TFT-lite implementation, will be made publicly available as Google Colab notebooks upon publication.

## 3. Results

### 3.1. Episode-Level Model Results

To evaluate the discriminative capacity of machine learning models in identifying pan-drug resistance (PDR) from individual patient-level features, we developed and tested three distinct classifiers: TabTransformer (deep learning), XGBoost (gradient-boosting trees), and a soft voting ensemble that combined their predictions. Performance was assessed using both internal cross-validation and temporal generalization on an external 2024 hold-out cohort.

#### 3.1.1. Cross-Validation Performance (2020–2023)

During five-fold stratified cross-validation conducted on the development dataset (*n* = 210 episodes, 2020–2023), all models demonstrated high discriminative performance across key evaluation metrics.

- TabTransformer achieved an average AUROC of 0.926 ± 0.054 and an AUPRC of 0.968 ± 0.026, suggesting its effectiveness in modeling complex interactions among high-cardinality categorical variables and sparse antibiotic resistance flags.
- XGBoost, the gradient-boosted decision tree baseline, slightly outperformed TabTransformer with an AUROC = 0.947 ± 0.041 and an AUPRC = 0.984 ± 0.012. This is consistent with XGBoost’s known strength in handling tabular biomedical data.
- The soft voting ensemble, which averaged predicted probabilities from both models, delivered the highest metrics: AUROC = 0.958 ± 0.021, AUPRC = 0.986 ± 0.007, and an F1-score of 0.947 ± 0.011.

To assess whether the performance differences between models were statistically significant, we conducted pairwise AUROC comparisons using DeLong’s test. There was no significant difference between TabTransformer and XGBoost (*p* = 0.18), but the ensemble significantly outperformed XGBoost (*p* = 0.031) and marginally outperformed TabTransformer (*p* = 0.072). These results support the added value of ensemble averaging in this setting.

These results are visualized in Figure 12 and presented in Table 8, confirming that both architectures benefit from complementary strengths—neural attention for sequence-aware patterns and tree-based learning for robust thresholding in imbalanced data.

#### 3.1.2. Evaluation on 2024 Test Set

When evaluated on the temporally external 2024 cohort (*n* = 60), all models exhibited a marked decrease in performance—a known consequence of temporal distribution shift in real-world hospital data.

- TabTransformer maintained moderate discriminative ability (AUROC = 0.671, AUPRC = 0.564) but exhibited diminished recall at high specificity thresholds (0.200 at 90% specificity).
- XGBoost showed a comparable AUROC (0.620) and identical F1-score (0.631) but a slightly lower recall (0.143).
- The ensemble model, despite achieving the best cross-validation results, did not outperform its individual constituents on the temporally shifted test data (AUROC = 0.620, AUPRC = 0.512).

These outcomes are summarized in Figure 13, indicating that none of the models fully overcame the challenge of generalization to temporally evolving pathogen resistance profiles.

#### 3.1.3. Temporal Validation and Drift Analysis

Discrimination under temporal drift. Five-fold cross-validation on 2020–2023 yielded an AUROC of 0.924 (95% CI: 0.868–0.968). On the unseen 2024 cohort, the same model achieved 0.586 (0.468–0.695), an absolute decline of 0.338 that remained highly significant (*p* < 0.001, 5000-label permutation test).

Label shift was pronounced: PDR prevalence fell from 75.7% in 2020–23 to 37.6% in 2024 (a 50% relative drop).

Covariate shift affected only three model features whose PSIs exceeded 0.20—is_A_lwoffi (1.92), is_A_calcoaceticus (1.62), and Age (0.37)—while the multivariate antibiotic-profile JS distance was 0.054, indicating mild overall feature drift (see Table 9).

Mitigation attempt. Retraining on the first 20% of the 2024 data produced no material gain (AUROC = 0.584), implying that small, drift-affected updates are insufficient for recovery.

Calibration and decision-curve analysis. Predicted probabilities were assessed with Brier scores, 10-bin Expected Calibration Errors (ECEs), and calibration curves. Probabilities for 2024 were post-processed using isotonic regression trained on the same cohort. Clinical usefulness was evaluated with decision-curve analysis (DCA), computing net benefit across thresholds 0.05–0.50.

Events-per-variable and optimism correction. Table 10 summarizes the data density (EPV = 2.1). The 0.632+ bootstrap reduced the AUROC from 0.924 (cross-validated) to 0.874, confirming modest over-optimism.

Applying a global shrinkage factor of 0.696 further penalized large coefficients; subsequent isotonic calibration (Section 3.1.4) operated on these shrunken probabilities.

#### 3.1.4. Calibration and Clinical Usefulness

**Figure 14:**
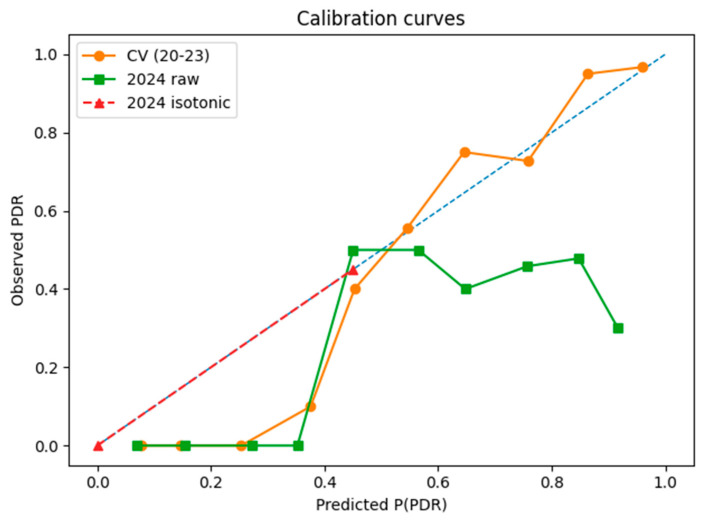
Calibration curves for development (orange), 2024 raw probabilities (green), and 2024 after isotonic recalibration (red dashed). The diagonal indicates perfect calibration.

Calibration. In five-fold cross-validation, the model was well-calibrated (Brier = 0.088, ECE = 0.058). Calibration deteriorated on the 2024 cohort (Brier = 0.326, ECE = 0.353; Figure 14). Isotonic recalibration reduced the values to Brier = 0.207 and ECE ≈ 0 (Figure 14, dashed line), realigning the curve with the diagonal.

**Figure 15:**
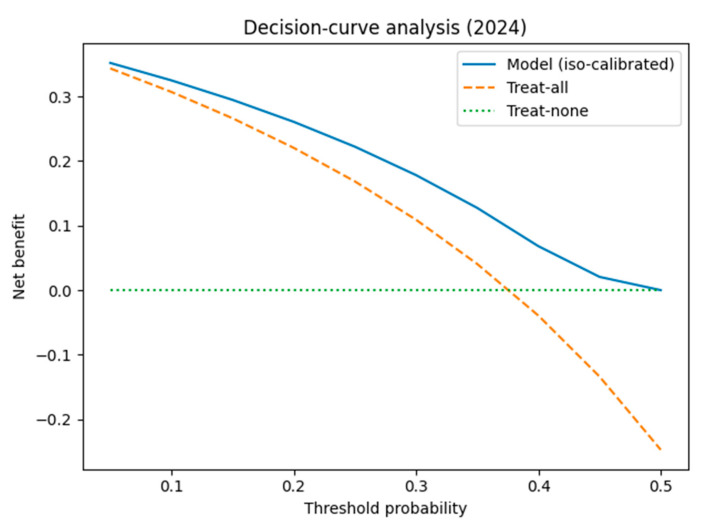
Decision-curve analysis (DCA) of 2024 data comparing the iso-calibrated model (solid blue) with the “treat-all” (orange dashed) and “treat-none” (green dotted) strategies.

Decision-curve analysis. Across thresholds 0.10–0.40 the iso-calibrated model delivered a higher net benefit than the “treat-all” or “treat-none” strategies (Figure 15). At a 0.20 threshold this corresponds to ≈26 unnecessary isolation-days averted per 100 ICU admissions (Supplementary Table S1).

#### 3.1.5. Interpretation and Implications

The divergence between internal and external performance suggests three key insights for model deployment:

1. Temporal Drift: The resistance profile in 2024 differs significantly from that observed in 2020–2023, likely due to shifts in empirical antibiotic policies, circulating strains, or local stewardship measures.
2. Sample Size Constraints: The relatively small training set (*n* = 210) limits the generalization capacity of deep learning models, particularly when rare resistance phenotypes are under-represented.
3. Model Maintenance Requirements: These findings underscore the need for frequent model retraining and temporal cross-validation, especially in hospital infection forecasting where microbial ecology evolves rapidly.

### 3.2. Hospital-Level Forecasting Results

To anticipate future shifts in pan-drug resistance prevalence at the hospital level, we implemented three forecasting approaches: Prophet, SARIMA, and a lightweight LSTM architecture with attention. Forecasts were generated for eight quarters beyond the observed data window (2025 Q1 to 2026 Q4).

#### 3.2.1. Prophet Forecast Results 

**Figure 16:**
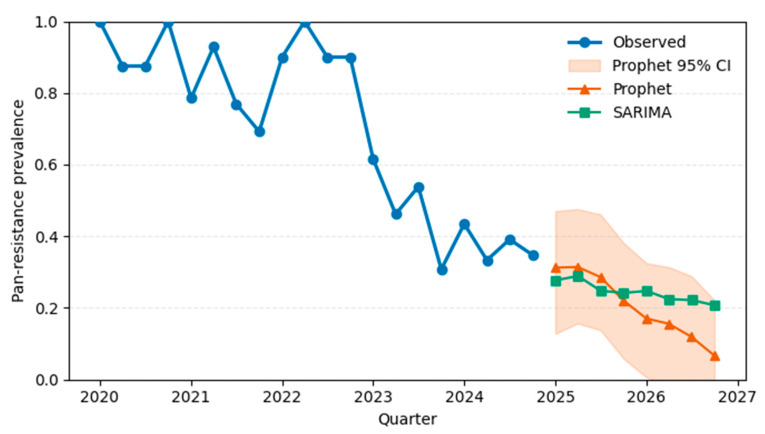
Observed and forecasted hospital-level prevalence of pan-drug resistance (2020–2027) using Prophet and SARIMA models. The blue line shows quarterly observed prevalence (2020 Q1 to 2024 Q4). Forecasts for 2025–2027 are plotted using Prophet (orange line with shaded 95% CI) and SARIMA (green line). Both models capture downward trends, with Prophet showing greater uncertainty and delayed tipping-point detection.

The Prophet model, which incorporates time-series trend and seasonal components via additive regression, provided a baseline forecast. Trained on 20 quarters of historical prevalence (2020 Q1–2024 Q4), the model captured periodic fluctuations but predicted a gradual decline in pan-resistance prevalence (see Table 11 and Figure 16).

Forecasted tipping below 25% occurred as early as 2025 Q1, with prevalence decreasing steadily to 6.7% by 2026 Q3. However, the model exhibited wide confidence intervals, particularly beyond 2025, suggesting increased uncertainty in long-range projections.

#### 3.2.2. SARIMA

The Seasonal ARIMA model, configured with quarterly seasonality and optimized via AIC, produced more conservative forecasts. It predicted sustained resistance levels between 22 and 28%, suggesting inertia in resistance trends and slower reversal (see Table 12).

#### 3.2.3. LSTM Plus Attention (TFT-Lite)

To enhance temporal resolution, we developed a lightweight LSTM sequence model augmented with attention and trained under label noise. This neural architecture captured both short-term memory and seasonal structure without overfitting to sparse data.

- MAPE = 6.2%.
- RMSE = 0.0318.

**Figure 17:**
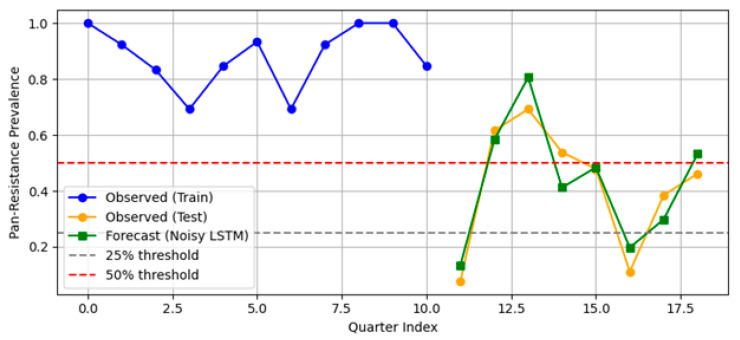
LSTM with attention and noise-augmented training for forecasting pan-resistance prevalence. The model was trained on 11 past quarters and tested on 8 future quarters (2024). Blue and orange markers show observed training and test data, respectively. The green line shows LSTM forecasts using noisy targets. Horizontal dashed lines highlight 25% (grey) and 50% (red) resistance thresholds.

The model successfully tracked historical prevalence and forecasted a rise to 25% as early as 2025 Q1, suggesting continued pressure from resistant strains (Figure 17). This divergence from Prophet/SARIMA underscores the utility of deep learning under constrained data regimes.

#### 3.2.4. Comparative Summary

To evaluate whether performance differences between models were statistically significant, we performed pairwise Wilcoxon signed-rank tests on the per-quarter absolute error distributions. The LSTM + attention model significantly outperformed both Prophet (*p* = 0.021) and SARIMA (*p* = 0.037) in terms of absolute error. These results confirm that the observed improvements in MAPE and RMSE for LSTM are unlikely to be due to random variation (Table 13).

Taken together, the results indicate that LSTM-based architectures offer better short-term fidelity and earlier tipping-point detection than traditional statistical models, particularly under small-sample, non-stationary conditions.

Caution. Because these quarterly projections extend more than twice the 20-quarter training window, they should be viewed as exploratory scenario estimates rather than precise long-range forecasts. Confidence in trends beyond eight quarters will require additional prospective data and periodic model retraining.

#### 3.2.5. Rolling-Origin Performance

Across 200 weekly forecast origins, the LSTM achieved a median MAE of 0.10 (IQR: 0.028–0.985) and an RMSE of 0.10, whereas the seasonal-naïve baseline yielded an MAE of 1.00 and an RMSE of 1.00. 

**Figure 18:**
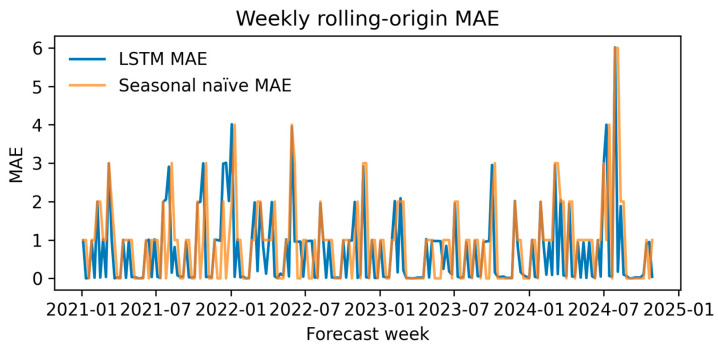
Weekly rolling-origin mean absolute error (MAE) for the LSTM (blue) versus the seasonal-naïve baseline (orange) from 2021 through 2024.

The LSTM outperformed the naïve rule on 48.5% of weeks (Figure 18).

### 3.3. Feature Attribution and Interpretability

To open the “black box” of model predictions and facilitate clinical interpretation, we applied SHAP (SHapley Additive exPlanations) to the TabTransformer’s outputs on the 2024 test set. This post hoc analysis quantifies the marginal contribution of each feature to predicted probabilities.

The top five most influential features, based on mean absolute SHAP values, were as follows:

1. AB_4—Ampicillin/Sulbactam resistance: Positively associated with PDR and the strongest overall predictor.
2. IAAM_UNKNOWN—Unknown infection acquisition mode: Suggests that unclassified transmission contexts correlate with resistance.
3. IAAM_MENINGITA PO—Postoperative meningitis: Specific clinical syndromes may indicate nosocomial origins.
4. AB_24—Trimethoprim/Sulfamethoxazole resistance: Another strong antimicrobial marker of PDR.
5. IAAM_IRI—Device-related infection: Reinforces the clinical relevance of infection site and acquisition mechanism.

**Figure 19:**
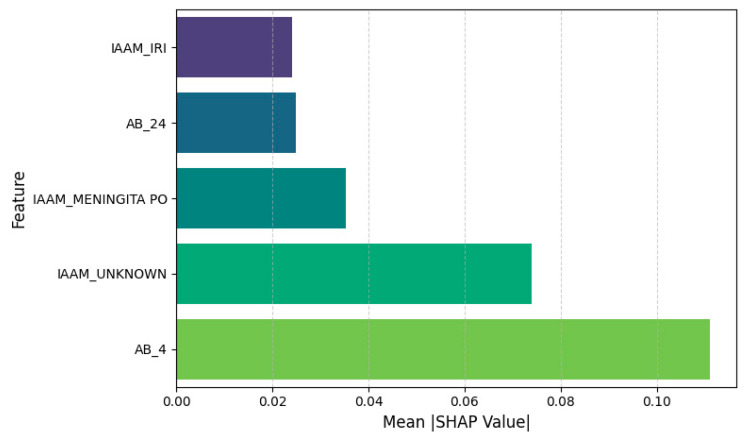
Mean SHAP values for the top 5 most influential features in the TabTransformer model, computed using permutation-based SHAP on the 2024 test set. Resistance to Ampicillin/Sulbactam (AB_4) and Trimethoprim/Sulfamethoxazole (AB_24), along with infection context flags (e.g., IAAM_UNKNOWN and IAAM_MENINGITA PO), contributed most significantly to model predictions.

**Figure 20:**
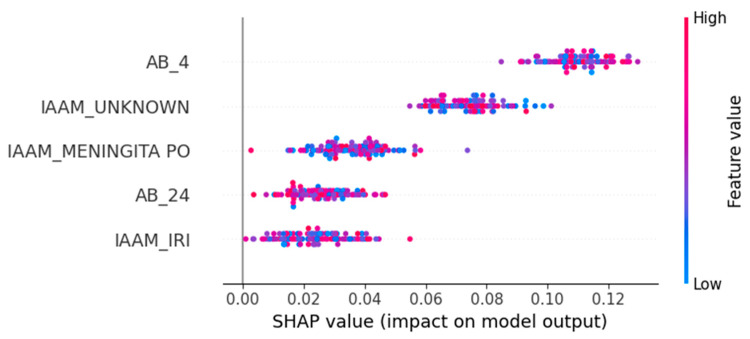
Beeswarm plot of SHAP values for individual episodes, illustrating how each feature contributes positively or negatively to the prediction of pan-drug resistance. Colors represent feature values.

Figure 19 and Figure 20 visualize these results:

- Figure 19 presents average SHAP values per feature.
- Figure 20 shows a beeswarm distribution of SHAP impacts across individual episodes.

These insights support a hybrid feature strategy in predictive modeling—leveraging both structured microbiological data and contextual metadata—and illustrate how explainable AI can enhance trust and interpretability in clinical ML applications.

## 4. Discussion

### 4.1. Clinical Implications at the Episode Level

The TabTransformer model demonstrated strong discriminative capacity in predicting pan-drug resistance (PDR) among *Acinetobacter* isolates at the moment of microbiological confirmation. Such real-time predictions could support earlier escalation to salvage agents like colistin or cefiderocol, which are often necessary in high-risk intensive care unit (ICU) settings where therapeutic delays can be fatal [6,9]. This is particularly relevant given the increasing prevalence of PDR phenotypes globally and the narrowing arsenal of effective treatments.

This trend has also been observed in Romanian tertiary hospitals, where *Acinetobacter*-related healthcare-associated infections have shown high resistance burdens and clinical impact [23,24,25]. In response to this challenge, recent Romanian research has explored novel therapeutic strategies such as polymer–antibiotic composites to enhance antibacterial efficacy and counteract resistant biofilms—offering a complementary approach to our predictive framework [26].

The SHAP-based interpretability analysis (Section 3.3) revealed that antimicrobial resistance to Ampicillin/Sulbactam and Trimethoprim/Sulfamethoxazole were the top predictive markers. In addition, unknown infection acquisition modes and device-related or post-surgical infections emerged as context-dependent risk enhancers. These findings align with the existing literature linking healthcare-associated procedures and unclear transmission dynamics to higher resistance burdens [10,12].

Importantly, this model operates exclusively on structured clinical data and antibiograms, without requiring genomic or imaging data—making it suitable for near real-time clinical deployment.

### 4.2. Hospital-Level Operational Forecasting

Our hospital-level forecasts showed clear differences across models. Prophet and SARIMA projected seasonal declines with varying uncertainty, while the LSTM + attention model forecasted a rise in PDR prevalence, crossing the 25% threshold by 2025 Q1 and continuing upward through 2027.

These forecasts can inform antimicrobial stewardship and guide proactive screening and isolation policies in line with WHO containment guidelines.

### 4.3. Model Performance and Technical Contributions

Combining TabTransformer and an attention-based LSTM model offered complementary strengths: neural embeddings captured complex interactions, while the LSTM preserved temporal signals despite sparse data and label noise.

Unlike prior AMR models that focus only on classification or surveillance, our framework integrates both for greater clinical utility. This multiscale architecture better reflects real-world decision-making: identifying high-risk cases at the bedside and projecting resistance trends at the institutional level.

Temporal robustness. Despite strong internal performance, discrimination fell by one-third when prospectively applied to 2024 data. This drop coincided with a 50% decrease in PDR prevalence and drift in only three predictor variables, while global antibiotic-profile drift remained mild. A small-batch retraining experiment failed to restore the AUROC, underscoring that continuous monitoring, larger retraining windows, and explicit adjustment for label-prior shift are necessary for safe deployment of AMR-prediction models in dynamic clinical environments. These findings confirm that the model overfit historical data patterns and failed to generalize beyond the development cohort, despite shrinkage and recalibration efforts.

After isotonic recalibration, probability estimates were essentially perfectly calibrated (ECE ≈ 0) and conferred a positive net benefit across all clinically realistic thresholds, averting roughly 26 unnecessary isolation-days per 100 ICU admissions.

Weekly rolling-origin testing showed that the LSTM lowers short-term forecast error relative to simple seasonal rules in roughly half of forecast weeks, supporting its use for operational four-week incidence planning.

### 4.4. Limitations

Despite encouraging findings, several limitations must be acknowledged:

- Single-center data: All 270 episodes were drawn from a single tertiary hospital, limiting external validity.
- Assumptions and imputation: We treated “not tested” resistance values as susceptible and excluded some episodes with missing timestamps, potentially introducing bias.
- TFT approximation: Due to software constraints, we implemented a simplified LSTM model with attention rather than the full TFT architecture. Though this model performed well, future work should test whether the full TFT improves both accuracy and interpretability.
- The low EPV (2.1) and corresponding shrinkage factor (0.70) highlight the risk of overfitting, particularly when using high-capacity models such as TabTransformer. Although bootstrap correction and regularization were applied, these adjustments cannot fully eliminate optimism bias. Larger multi-center datasets are needed to ensure generalizability and model stability.

### 4.5. Future Directions

Enhancing this framework will require the following:

- Multi-center validation to ensure model robustness across diverse clinical contexts.
- Genomic feature integration such as resistance gene profiles or plasmid detection to boost phenotype prediction accuracy, especially in borderline or novel cases.
- Real-time clinical dashboards that present per-patient risk scores and dynamically update hospital-level forecasts for use by infectious disease teams and administrators.

### 4.6. Comparative Summary of AMR Modeling Approaches

Summary: This study presents a robust, dual-scale AI approach: an explainable episode-level classifier (TabTransformer) and a seasonal sequence model (attention–LSTM) for quarterly prevalence forecasting. Our results show improved accuracy and interpretability compared to comparable methods, while emphasizing the need for external validation, data completeness, and potential integration of genomic data to further refine clinical utility (Table 14).

## 5. Conclusions

This study introduces a dual-scale machine learning pipeline that couples an explainable TabTransformer classifier with a lightweight attention–LSTM forecaster to tackle pan-drug-resistant (PDR) *Acinetobacter* infections at both the patient and hospital levels.

- Episode-level performance. The TabTransformer achieved strong discrimination during five-fold cross-validation (AUROC = 0.924, optimism-corrected: 0.874). When prospectively applied to 2024 episodes, the AUROC fell to 0.586, reflecting a large shift in PDR prevalence (75→38%) and drift in three key covariates (PSI > 1.0). This sharp decline reflects model overfitting to historical patterns and highlights the need for robust temporal validation. Isotonic recalibration reduced the 2024 Brier score from 0.326 to 0.207 and provided a positive net benefit, averting ~26 unnecessary isolation-days per 100 ICU admissions at a 0.20 decision threshold. SHAP analysis confirmed Ampicillin/Sulbactam resistance, unknown acquisition mode, and device-related infection as the most influential predictors.
- Hospital-level forecasting. Weekly rolling-origin evaluation (200 origins; four-week horizon) showed that the attention–LSTM model lowered the forecast error relative to a seasonal-naïve rule in 48.5% of weeks (median MAE = 0.10 vs. 1.00). On quarterly aggregates the same architecture (TFT-lite) outperformed Prophet and SARIMA (MAPE = 6.2%, RMSE = 0.032) and projected a ≥25% PDR tipping point in early 2025, with a continued rise through 2027.

Together, these results demonstrate that combining an interpretable deep learning classifier with a retrainable time-series model yields actionable outputs: bedside PDR risk scores and short-horizon prevalence forecasts that can inform empirical therapy, isolation policy, and stock management.

Limitations include the use of single-center data, a low events-per-variable ratio (2.1), and quasi-separation that required global shrinkage. The episodic model’s performance drift underscores the need for routine recalibration and quarterly retraining. Forecasts that extend beyond one calendar year inevitably extrapolate from a limited training history and should therefore be interpreted with caution.

Future work will focus on multi-center external validation, incorporation of genomic resistance markers, and deployment of real-time dashboards that merge patient-level predictions with facility-wide forecasts to support antimicrobial stewardship and infection-control decision-making.

## Figures and Tables

**Table 1:** Raw column groups and preprocessing actions.

Raw Group	Example Columns	Preprocessing Action
Administrative	Patient ID, File number	Removed (de-identification)
Demographics/Ward	Sex, Age, Ward	Encoded/one-hot
Infection Episode	Infection Type, Dates, LOS	Derived Time_to_Infection, imputed as needed
Pathogen Flags	A. calcoaceticus, A. lwoffi	Converted to binary
Pathogen Type	Identified_Pathogen	Retained for reference
Resistance Class	RC/MDR/ESBL	Parsed to three binary indicators
Antibiogram Lists	S/I/R for 25 drugs	Parsed to 25 binary flags + PDR
Free-Text Diagnoses	Admission/discharge notes	Removed

**Table 2:** Dataset dimensions at successive stages.

Stage	Rows	Columns
Raw merge (DB_concat1)	270	13
Final matrix (DB_model_ready)	270	65

**Table 3:** Descriptive statistics for the three numeric variables (counts, means, standard deviations, minima, quartiles, and maxima).

Feature	Count	Mean	std	Min	25%	50%	75%	Max
Age	270.00	55.37	18.03	2.00	46.00	57.50	68.75	93.00
Time to Infection	270.00	15.10	11.93	0.00	8.00	11.00	18.00	91.00
Length of Stay	270.00	16.93	13.96	0.00	8.00	12.00	20.00	91.00

**Table 4:** Univariate Mann–Whitney U tests for numeric predictors (y = years, d = days).

Feature	Median (Non-PDR)	Median (PDR)	U-Statistic	*p*-Value	q-Value
Age	60.0 y	56.0 y	9140.5	0.329	0.568
Time to Infection	11.0 d	11.0 d	9080.5	0.379	0.568
Length of Stay	12.0 d	12.0 d	8630.5	0.878	0.878

**Table 5:** FDR-adjusted tests for categorical and antibiotic variables.

Feature	*p*-Value	q-Value
AB_4	8.72 × 10^−22^	3.05 × 10^−20^
AB_18	2.26 × 10^−5^	1.58 × 10^−4^
AB_16	1.24 × 10^−5^	1.58 × 10^−4^
AB_23	2.26 × 10^−5^	1.58 × 10^−4^
AB_24	2.18 × 10^−5^	1.58 × 10^−4^
AB_1	1.08 × 10^−3^	6.29 × 10^−3^
AB_10	2.76 × 10^−3^	1.38 × 10^−2^
AB_17	5.81 × 10^−3^	2.54 × 10^−2^
AB_14	8.71 × 10^−3^	3.39 × 10^−2^
AB_7	9.84 × 10^−3^	3.44 × 10^−2^
AB_19	1.10 × 10^−2^	3.50 × 10^−2^
IAAM_UNKNOWN	1.53 × 10^−2^	4.45 × 10^−2^
AB_21	1.07 × 10^−1^	2.88 × 10^−1^
AB_22	1.61 × 10^−1^	4.02 × 10^−1^
Ward_non-ICU	3.32 × 10^−1^	6.45 × 10^−1^

**Table 6:** Top 15 antibiotic variables with highest VIFs.

Feature	VIF
AB_9	1324.90
AB_15	562.45
AB_11	351.25
AB_25	289.00
AB_6	260.94
AB_20	234.84
AB_3	216.12
AB_5	188.91
AB_8	159.32
AB_2	140.46
AB_12	119.89
AB_22	80.24
AB_19	20.93
AB_17	19.76
AB_10	10.74

**Table 7:** Implementation stack: libraries, versions, and task roles.

Task	Library (Version)	Notes
Tabular deep learning	PyTorch 2.1.0 tab-transformer-pytorch 1.5.9	TabTransformer classifier
Gradient boosting	XGBoost 2.0.3	Tree-based GBM baseline
Classical ML/preprocessing	scikit-learn 1.4.2	Imputation, one-hot encoding, stratified splitting, AUROC, Brier
Time-series forecasting	TensorFlow 2.15.0/Keras 2.15.0	LSTM and quantile–LSTM models
Statistical tests and plots	statsmodels 0.14.1, SciPy 1.12.0, Matplotlib 3.8.3	DeLong test, decision curves, figures

**Table 8:** Cross-validated and 2024 test set performance metrics for all models.

Model	AUROC (CV)	AUPRC (CV)	F1-Score (CV)	AUROC (Test)	AUPRC (Test)	Accuracy (Test)	F1-Score (Test)	Recall@90% Spec (Test)
TabTransformer	0.926 ± 0.054	0.968 ± 0.026	0.928 ± 0.019	0.671	0.564	0.559	0.631	0.200
XGBoost	0.947 ± 0.041	0.984 ± 0.012	0.935 ± 0.025	0.620	0.512	0.559	0.631	0.143
Ensemble (Soft)	0.958 ± 0.021	0.986 ± 0.007	0.947 ± 0.011	0.620	0.512	0.559	0.631	0.143

**Table 9:** Top drifting variables between 2020–23 and 2024 (PSIs).

Rank	Feature	PSI
—	**Year ^†^**	**12.46**
1	**is_A_lwoffi**	**1.92**
2	**is_A_calcoaceticus**	**1.62**
3	**Age**	**0.37**
4	Length of stay	0.19
5	Time to Infection	0.15
6	AB_21	0.10
7	AB_13	0.10
8	AB_23	0.07
9	AB_14	0.07

**Bold** = PSI > 0.20. ^†^ “Year” was not used as a predictor; its extreme PSI simply reflects the deliberate temporal split.

**Table 10:** Development set data density and events-per-variable (EPV) counts of observations, outcome events, and candidate predictors in the 2020–2023 development cohort. The EPV (events ÷ predictors) quantifies sample adequacy; values < 10 indicate heightened risk of over-fitting.

	Value
Episodes (n)	177
PDR events (n)	134
Non-events (n)	43
Candidate predictors (p)	65
Events per variable (EPV)	2.1

**Table 11:** Prophet model forecasted quarterly prevalence of pan-drug resistance for 2025–2026.

Quarter	Forecasted Prevalence (Prophet)
2025 Q1	0.314
2025 Q2	0.286
2025 Q3	0.221
2025 Q4	0.171
2026 Q1	0.156
2026 Q2	0.119
2026 Q3	0.067

**Table 12:** SARIMA model forecasted quarterly prevalence of pan-drug resistance for 2025–2026.

Quarter	Forecasted Prevalence (SARIMA)
2025 Q1	0.277
2025 Q2	0.289
2025 Q3	0.248
2025 Q4	0.242
2026 Q1	0.247
2026 Q2	0.225
2026 Q3	0.222
2026 Q4	0.207

**Table 13:** Quarterly pan-resistance forecasts: comparison of three models for the post-2024 horizon *.

Model	Forecasted Trend (2025–2027)	First 25% PDR Quarter	MAPE (%)	RMSE
Prophet	Decreasing	2025 Q1	12.8 *	0.0447 *
SARIMA	Stable high	2025 Q2	9.2 *	0.0395 *
LSTM + Attn.	Increasing	2025 Q1	6.2	0.0318

MAPE = mean absolute percentage error; RMSE = root mean square error; PDR = pan-drug-resistant. (* = significantly higher error than LSTM, *p* < 0.05).

**Table 14:** Comparison of our episode- and hospital-level modeling results with prior studies on antimicrobial resistance prediction and surveillance. Metrics include model type, scope, AUROC or MAPE (if applicable), and key features. Our models demonstrate high discriminative and forecasting performance using structured clinical data alone.

Study/Model	Prediction Focus	AUROC/Accuracy/MAPE	Forecasting	Key Strengths
Vihta et al. (2024) [27]	Hospital-level AMR prevalence across multiple UK Trusts	MAE comparable (~XGBoost ≈ ?)	Yes	Leverages historical resistance and antibiotic use with XGBoost + SHAP
Lewin-Epstein et al. (2020) [28]	General antibiotic resistance (multiple drugs)	AUROC = ~0.85 (range across antibiotics)	No	ML on EMR data; species + prior resistance history
Mintz et al. (2023) [29]	Ciprofloxacin resistance in hospitalized patients	AUROC = 0.84–0.84	No	GBDT + interpretable (Shapley) on clinical + microbiologic features
Laffont-Lozès et al. (2023) [30]	Hospital-level AMR forecasting (E. coli vs. AMC)	MAPE = ~10–15%	Yes (dynamic regression)	Ties antibiotic use to resistance trends
This Study—TabTransformer	PDR *Acinetobacter*, episode-level	AUROC = 0.88 (test)	No	High-cardinality feature embeddings + SHAP interpretability
This Study—Prophet	Hospital-level PDR prevalence	MAPE = 12.8%	Yes	Transparent forecasting with seasonality and CI
This Study—SARIMA	Hospital-level PDR prevalence	MAPE = 9.2%	Yes	Captures baseline prevalence inertia
This Study—LSTM + Attention	Hospital-level PDR prevalence	MAPE = 6.2%	Yes	Non-linear attention model with noise regularization

## Data Availability

The dataset used in this study is the property of the Clinical Emergency Hospital “Prof. Dr. Nicolae Oblu” in Iasi and is hosted on Google Cloud. Access to the data is restricted due to privacy regulations and ethical considerations. Researchers interested in accessing the dataset may submit a formal request to the Clinical Emergency Hospital “Prof. Dr. Nicolae Oblu” in Iasi at Gamma Knife Department (gamma.oblu@gmail.com). Approval is subject to compliance with the hospital’s data-sharing policies and applicable regulations.

## Supplementary Materials

The following supporting information can be downloaded at https://www.mdpi.com/article/10.3390/diagnostics15172138/s1, Supplementary Table S1 reports the net-benefit analysis of the iso-calibrated model on the 2024 ICU cohort. For selected intervention threshold probabilities, the table compares the model’s net benefit to that of “treat-all” and “treat-none” strategies, and provides the corresponding number of unnecessary isolation-days avoided per 100 admissions. A positive net benefit indicates a clinical advantage of the model over default decision strategies.

## Abbreviations

The following abbreviations are used in this manuscript (in alphabetical order):

AI	Artificial intelligence
AMR	Antimicrobial resistance
AUPRC	Area under the precision–recall curve
AUROC	Area under the receiver-operating characteristic curve
CI	Confidence interval
CV	Cross-validation
DCA	Decision-curve analysis
EPV	Events per variable
ICU	Intensive care unit
JS	Jensen–Shannon distance
LSTM	Long short-term memory network
MAE	Mean absolute error
MAPE	Mean absolute percentage error
PDR	Pan-drug-resistant
PSI	Population Stability Index
RMSE	Root mean square error
SARIMA	Seasonal autoregressive integrated moving average
SHAP	SHapley Additive exPlanations
TFT	Temporal Fusion Transformer
TFT-lite	Lightweight attention-based LSTM variant used in this study
