# Supplementary material for: Episode- and Hospital-Level Modeling of Pan-Resistant Healthcare-Associated Infections (2020–2024) Using TabTransformer and Attention-Based LSTM Forecasting
Source: Diagnostics (Basel). 2025 Aug 25;15(17):2138. doi: 10.3390/diagnostics15172138 (PMC12428759; doi:10.3390/diagnostics15172138)
Supplement: Supplementary file 1 [file diagnostics-15-02138-s001.zip › diagnostics-3802494-supplementary.pdf]

Supplementary Tables

Supplementary Table S1. Net-benefit analysis of the iso-calibrated model on the 2024 cohort

Threshold	Net benefit (model)	Net benefit (treat-all)	ΔIsolation-days / 100 pts
0.20	0.261	0.114	**+26.1**

The table reports, for each intervention threshold probability, the model's net benefit, the net benefit of "treat-all" and "treat-none" strategies, and the implied number of unnecessary isolation-days avoided per 100 ICU admissions. Net benefit is calculated as

$$NB = \frac{TP}{N} - \frac{FP}{N}\left(\frac{t}{1-t}\right)$$

where *TP* = true positives, *FP* = false positives, *N* = cohort size, and *t* = threshold probability. Positive values indicate a clinical advantage over both default strategies.
